# Feasibility of fine needle aspiration for diagnosis of b-cell lymphoma of the thyroid: a case series and review of the literature

**DOI:** 10.1186/s13000-023-01346-4

**Published:** 2023-05-18

**Authors:** Alexander D. Karabachev, William J. Brundage, Mirabelle B. Sajisevi, Allison L. Ciolino

**Affiliations:** 1 Department of Otolaryngology, University of Cincinnati Medical Center, Cincinnati, OH USA; 2 Robert Larner College of Medicine, University of Vermont, Burlington, VT USA; 3 Department of Surgery, Division of Otolaryngology, University of Vermont Medical Center, Burlington, VT USA; 4 Department of Pathology and Laboratory Medicine, University of Vermont Medical Center, Burlington, VT USA

**Keywords:** Pathology, Thyroid cancer-clinical, Thyroid lymphoma, Fine needle aspiration, Cell block, Diagnosis

## Abstract

**Background:**

Primary thyroid lymphoma (PTL) is a rare cancer accounting for approximately 5% of thyroid malignancies. Historically, incisional biopsy has been the gold standard for definitive diagnosis of PTL, however, the use of cell block as an adjunct to fine needle aspiration (FNA) provides a high sensitivity and specificity for diagnosis and classification.

**Methods:**

Three patients presented with a symptomatic enlarging thyroid mass. Patient 1 underwent incisional biopsy under general anesthesia, Patient 2 underwent core needle biopsy to avoid high risk intubation, and Patient 3 underwent fine needle aspiration alone with the use of cell block.

**Results:**

All patients were diagnosed with a fully classified non-Hodgkin’s lymphoma using immunohistochemistry, flow cytometry, and fluorescence in situ hybridization (FISH) analysis.

**Conclusions:**

FNA for diagnosis of some subtypes of PTL is feasible and preferred in cases that are particularly high risk for general anesthesia. This minimally invasive technique is safe and cost effective as it avoids expenses associated with operative intervention.

## Introduction

Primary thyroid lymphoma (PTL) is a rare cancer accounting for approximately 5% of thyroid malignancies and 2% of all extranodal lymphomas [1]. Histologically, most are non-Hodgkin’s lymphoma (NHL) of B-cell origin and commonly affect women over the age of sixty years old [2]. The most common subtype of PTL is diffuse large B-cell lymphoma (DLBCL) typically arising in the setting of Hashimoto thyroiditis [3]. Greater than 50% of thyroid lymphomas are attributed to DLBCL while mucosa-associated lymphoid tissue (MALT) has been cited to account for 10% to up to 50% of cases [4]. Other less common lymphomas of the thyroid that have been described include Burkitt lymphoma, Hodgkin lymphoma, mantle cell lymphoma, T-cell lymphoma, follicular lymphoma and small lymphocytic lymphoma. [5]. Hashimoto thyroiditis may cause a 40-to-80-fold increase in the risk of developing PTL [4]. A review published in 2020 of 1,346 cases of PTL revealed 78.9% of these patients have evidence of Hashimoto thyroiditis [5].

Historically, incisional biopsy has been the gold standard for the diagnosis of NHL, however, a cytologic approach has gained acceptance at many institutions [6]. The ability to subclassify NHL with fine needle aspiration (FNA) based on the World Health Organization classification of lymphoid malignancies is largely due to ancillary studies performed on cytology material including flow cytometry, fluorescent in-situ hybridization (FISH), and immunohistochemical (IHC) staining. The avoidance of incisional biopsy and the utilization of FNA is safer and more cost effective yet its role in definitive diagnosis and classification of lymphoma remains controversial [7–10].

In the last decade, the use of cell blocks as an adjunct to FNA has been shown to provide high sensitivity and specificity for the diagnosis and classification of lymphoma. This method may be utilized to provide additional sample for IHC and other ancillary testing as well as the ability to assess improved tissue architecture without the need for core needle or surgical excision [9]. Here we report the diagnosis of thyroid lymphoma using FNA, core needle biopsy (CNB) and incisional biopsy to highlight the various methods of diagnosis and to demonstrate the use of FNA with cell block for definitive diagnosis and classification.

## Processing of FNA or surgical tissue

FNA was performed using a 25-gauge needle with or without suction. Rapid on-site evaluation (ROSE) was performed at the time of FNA by a board-certified cytopathologist or cytopathology fellow to provide immediate cytologic interpretation and appropriately triage material for ancillary testing. A small amount of aspirated material was expelled onto two slides; one of which was smeared and immediately placed into Coplin jars containing 95% ethanol for fixation, while the other slide was smeared and allowed to air dry. Needle rinses were performed in Roswell Park Memorial Institute (RPMI) medium. Additional needle passes were performed and placed entirely into RPMI for flow cytometry or cell block preparation. The slides and RPMI were immediately brought to the cytology laboratory for processing. Following processing, the alcohol-fixed slides were stained with the Papanicolaou stain and air-dried slides were stained with the May-Grunwald Giemsa stain. Air-dried preparations needed for potential FISH analysis were not stained and sent to cytogenetics. If indicated, a cell block was prepared by centrifuging the RPMI fluid for 5 min at 2000pm, decanting all but 0.25-0.5 cc of supernatant, and adding plasma and Thromboplastin-DA (Pacific Hemostasis™) to the cell button to form a clot. The clot was then placed on formalin-soaked filter paper which was folded and placed into a cassette for fixation in 10% buffered formalin for a minimum of 6 h followed by processing in histology where the tissue was ultimately embedded in a paraffin wax for sectioning.

Surgical tissue from CNB, typically performed with a 16- or 18-gauge needle, was placed into 10% formalin for transport to surgical pathology for grossing and then processed in histology. Larger tissue samples were sent fresh, in normal saline, to surgical pathology for lymphoma work-up and approximately one cubic centimeter of tissue was placed into RPMI for flow cytometry. Touch preparations from fresh tissue were allowed to air dry for potential FISH studies. The remaining tissue was sectioned, placed into cassettes, and fixed in 10% buffered formalin for a minimum of 6 h. Following fixation, the tissue was processed, embedded, sectioned at 5 microns, and mounted onto glass slides in histology.

## Results

### Patient 1: incisional biopsy

**Fig. 1:**
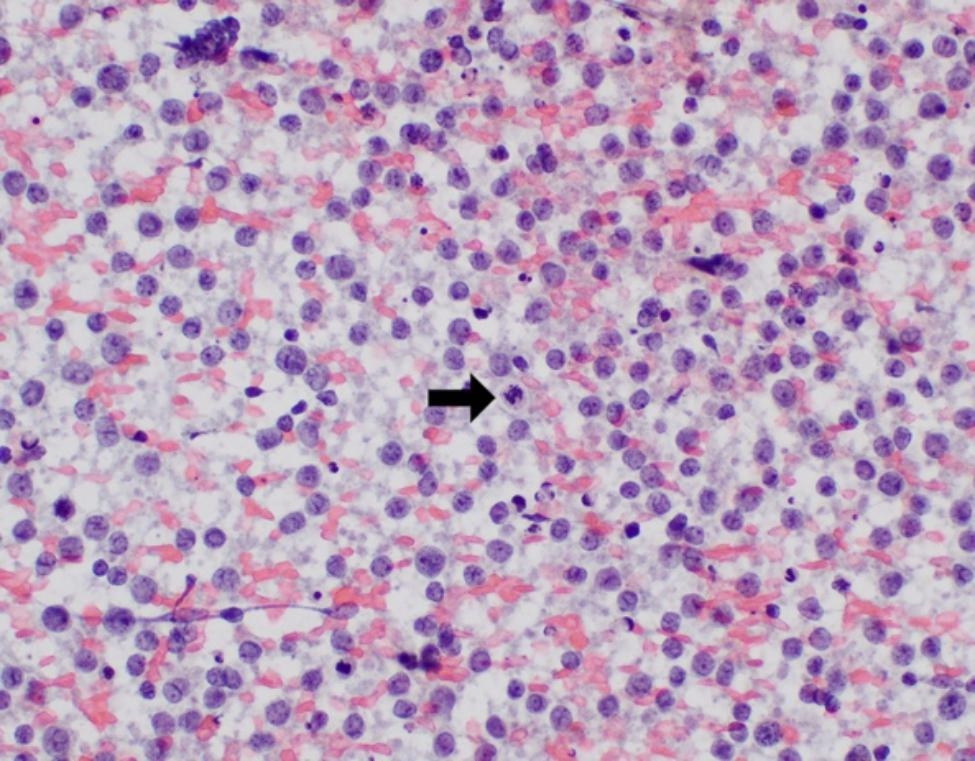
FNA of thyroid nodule (Case 1) showing an abnormal population of intermediate size lymphocytes in a background of abundant necrosis, apoptosis, and mitosis (arrow) (Pap stain, 400x magnification)

Patient is a 61-year-old transgender female with a history of Hashimoto’s thyroiditis with progressive enlargement of her thyroid for one year. Computed tomography (CT) scan revealed an 8.0 cm hypodense mass in the lower pole of the left thyroid lobe resulting in mild narrowing of the trachea. Ultrasound-guided FNA with ROSE was performed and material was obtained for flow cytometry and cell block due to concern for possible lymphoma. FNA revealed a monomorphic population of intermediate to large size lymphocytes with round to irregular nuclear contours, vesicular chromatin, distinct nucleoli, and inconspicuous cytoplasm (Fig. 1).

**Fig. 2:**
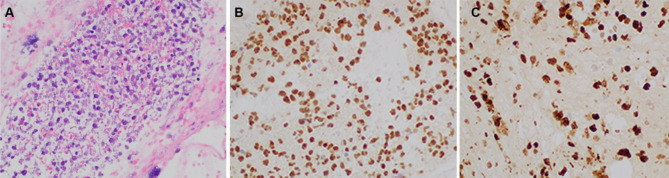
Cell block (Case 1) demonstrating abnormal lymphocytes with associated necrosis and apoptotic debris (A) (H&E stain, 400x magnification). Pax-5 IHC shows strong nuclear staining (B) in majority of lymphocytes supporting B-cell origin (B) (400x magnification). Ki-67 IHC shows positive nuclear staining in 80–90% of tumor cells (C) (400x magnification)

A cell block was prepared and showed similar tumor cells with adequate material for IHC (Fig. 2).

Flow cytometry studies revealed a CD10 positive and lambda light chain restricted population of B-cells. IHC performed on the cell block showed positive staining for CD20 (L26, Ventana), PAX-5 (1EW, Leica), Ki67 (MIB-1) (K2, Leica) in 80–90% cells and negative staining for CD3 (SP7, Thermo Scientific), Myc (Y69, Abcam), BCL-2 Oncoprotein (124, Ventana), EBER ISH (ISH5687-A, Leica) and CD138 (M115, Leica); indicating a large B-cell lymphoma with a differential diagnosis of high grade B-cell lymphoma versus DLBCL. FISH analysis for *MYC* rearrangement was indicated to complete the classification. Air dried smears from the FNA for FISH were inadvertently stained, thus additional material was needed. The patient was taken to the operating room for incisional biopsy under general anesthesia. Although intubation was difficult, her airway was ultimately secured, and she tolerated the procedure without complications.

**Fig. 3:**
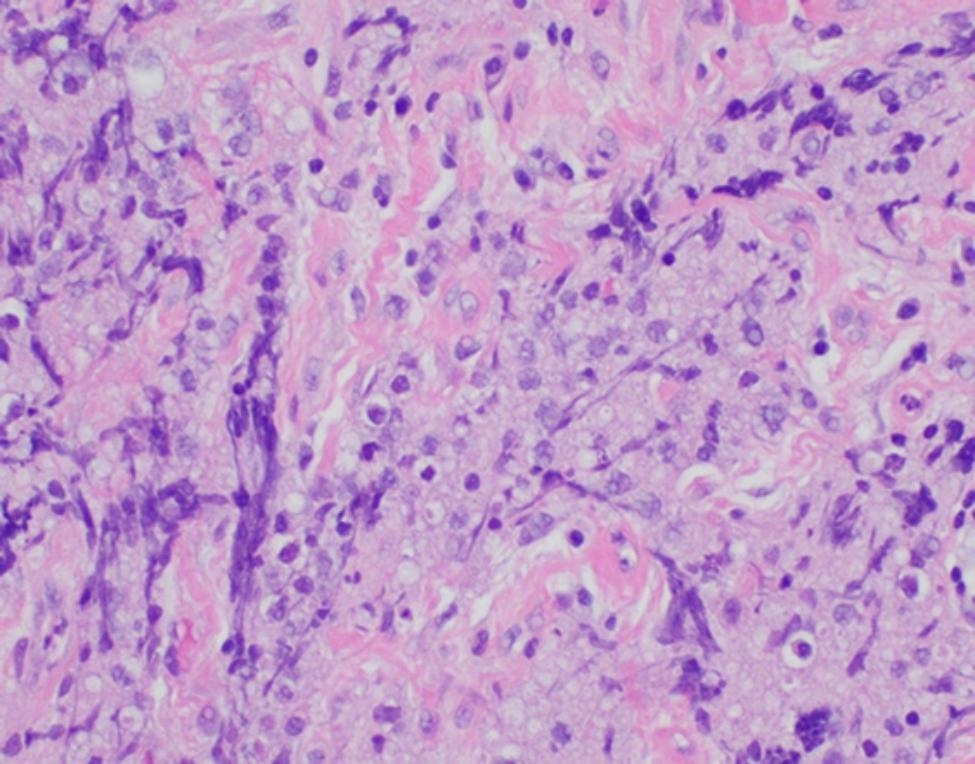
Incisional biopsy (Case 1) with viable tumor cells (right) and extensive crush artifact (left) (H&E stain, 400x magnification)

The incisional biopsy of the left thyroid mass showed similar neoplastic cells seen in the prior FNA arranged in a diffusely infiltrating pattern with intervening fibrous stroma and extensive crush artifact (Fig. 3).

Concurrent flow cytometry studies again revealed a clonal CD10 + lambda restricted B-cell population and IHC showed similar results to the prior studies performed on the cell block. In addition, the tumor cells were positive for BCL-6 (G/191E/A8) andCD10 (SP67, Ventana) and negative for Cyclin D1 (SP4-R, Ventana) and MUM-1 (MUM1p, Dako). No rearrangement or fusion of *MYC* were observed by FISH analysis. The diagnosis of DLBCL, germinal center type, was confirmed.

### Patient 2: core biopsy

**Fig. 4:**
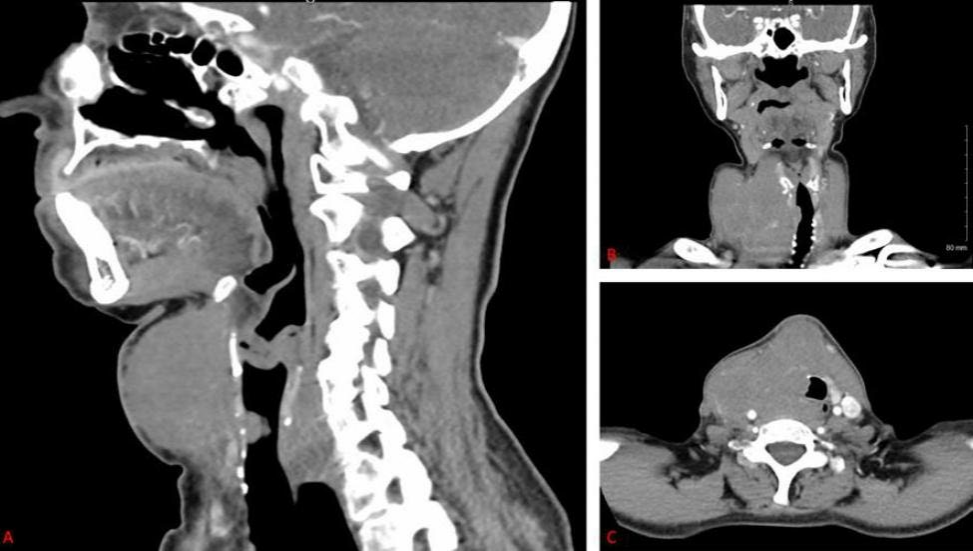
Sagittal (A), coronal (B), and axial (C) images from a computed tomography of the neck revealing an 8-centimeter mass within the thyroid gland with destruction of the thyroid cartilage and evidence of narrowing of the cervical trachea

Patient is a 69-year-old cis female with a history of hypothyroidism secondary to Hashimoto thyroiditis and long-standing multinodular goiter who presented to the Otolaryngology clinic for evaluation of an enlarging thyroid mass over 3 months. She had hoarseness, dysphagia, and right vocal cord paresis. CT of the neck revealed an 8.0 cm mass within the thyroid with destruction of the thyroid cartilage and evidence of narrowing of the cervical trachea (Fig. 4).

**Fig. 5:**
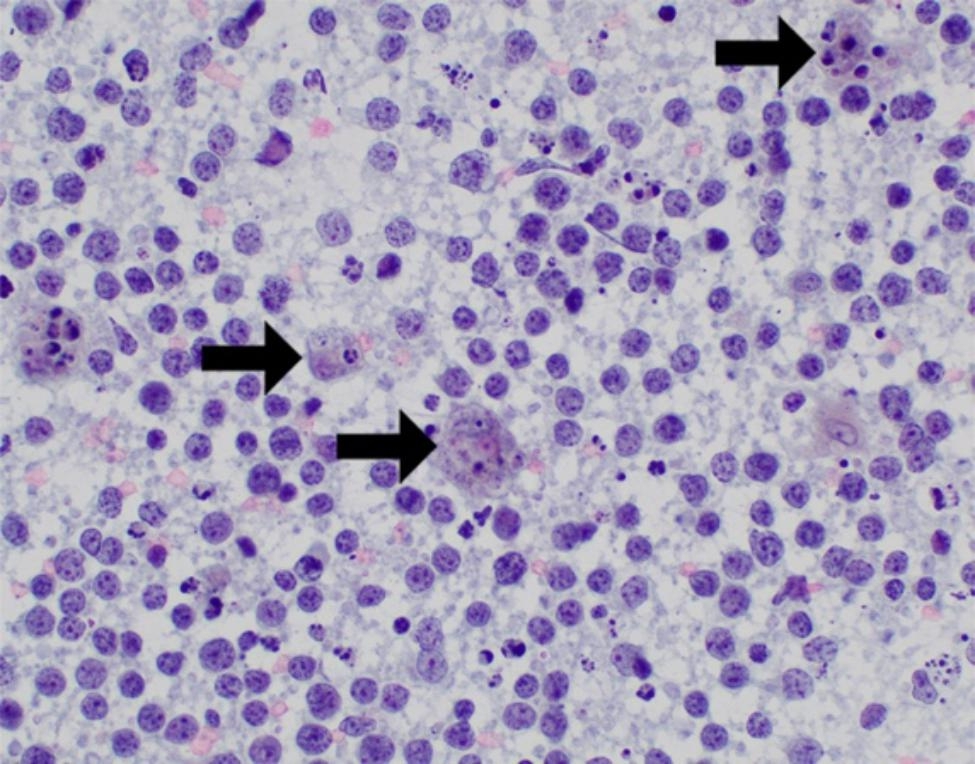
FNA of thyroid nodule (Case 2) with a population of large abnormal lymphocytes in a background of

Ultrasound-guided FNA with ROSE revealed a neoplastic proliferation of large lymphocytes with round to irregular nuclear contours, occasional nucleoli, and scant cytoplasm. Mitotic figures, apoptotic bodies and tingible-body macrophages were readily identified (Fig. 5).

tingible-body macrophages (arrows), necrosis and apoptosis (Pap stain, 400x magnification).

Material was obtained for flow cytometry which demonstrated a clonal B-cell population that was negative for CD10 and surface light chain expression; however, material was not obtained for cell block and therefore IHC could not be performed. The cytologic features in conjunction with results of flow cytometric analysis was diagnostic of large B-cell lymphoma. Additional tissue was required for further classification and prognostic purposes including FISH studies for *MYC* rearrangements.

**Fig. 6:**
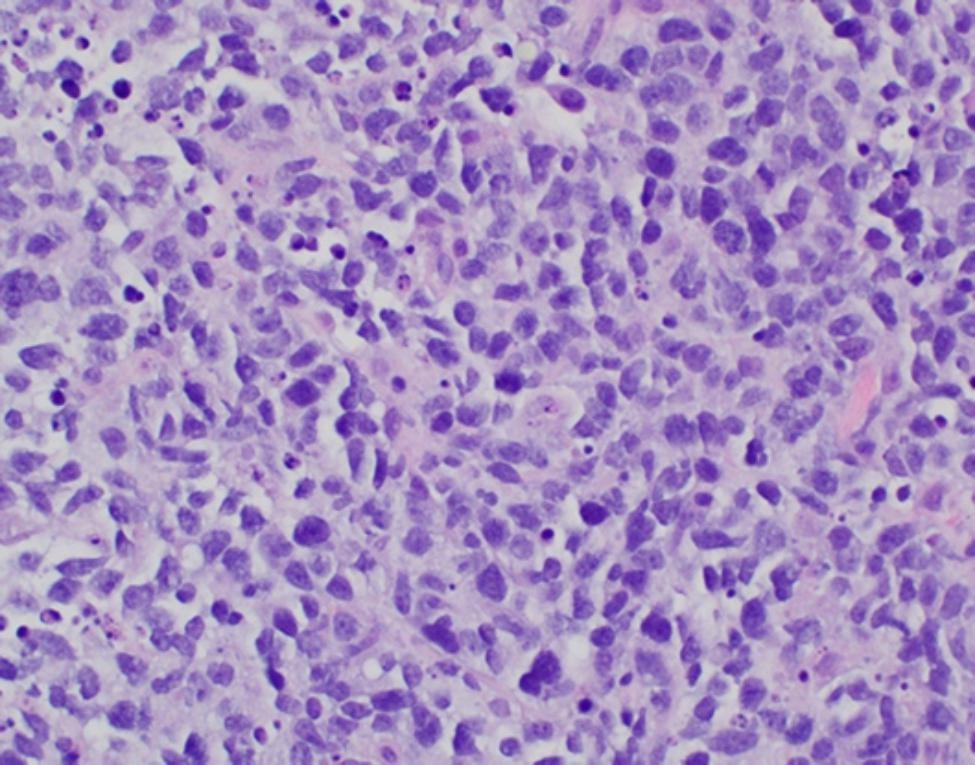
Core biopsy (Case 2) with large irregular lymphocytes and apoptosis (H&E stain, 400x magnification)

Given concern for high-risk intubation due to presence of tracheal deviation and invasion, decision was made to proceed with CNB. The biopsy revealed a diffuse pattern of similar large B-cells to those seen in the prior FNA (Fig. 6) and repeat flow cytometry also showed similar findings to those seen previously except dim lambda light chain expression was now discernable.

**Fig. 7:**
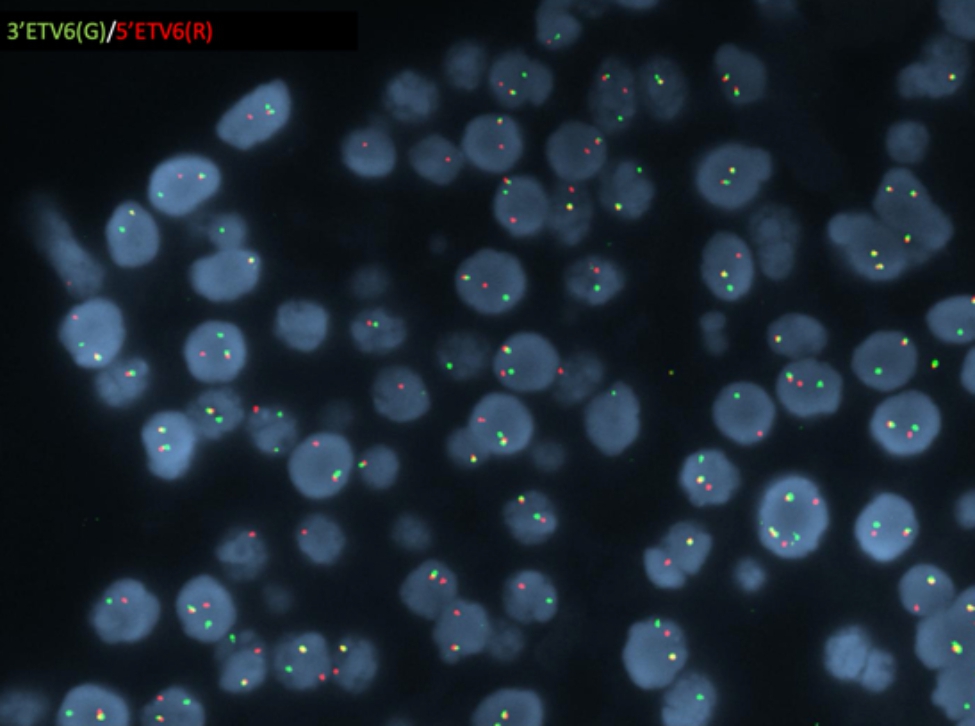
Fluorescence in situ hybridization analysis demonstrating the MYC/IGH gene fusion

IHC was positive for CD20 (L26, Ventana), BCL-6 (G/191E/A8) MYC (Y69, Abcam), Ki67 (MIB-1) (K2, Leica) in approximately 100% of cells and negative for CD3 (SP7, Thermo Scientific), CD10 (SP67, Ventana), BCL-2 Oncoprotein (124, Ventana), MUM-1 (MUM1p, Dako) and EBER ISH (ISH5687-A, Leica). FISH study for *MYC* gene rearrangement, performed at Mayo Medical Laboratories, was positive for *MYC/IGH* gene fusion (Fig. 7).

The *BCL-2* and *BCL-6* genes were germline. Based on these findings the diagnosis of high-grade B-cell lymphoma, not otherwise specified, was rendered.

### Patient 3: fine needle aspiration

Patient is a 69-year-old cis female with a history of Hashimoto’s hypothyroidism who presented to the emergency department for rapidly increasing left sided neck swelling. CT of the neck showed a 2.3 cm left thyroid nodule with an adjacent necrotic lymph node. She was treated with Augmentin which reduced the swelling significantly, however, continued to have a tender nodule in the left thyroid. Two months later, repeat CT scan demonstrated progression of the left cervical necrotic lymphadenopathy.

**Fig. 8:**
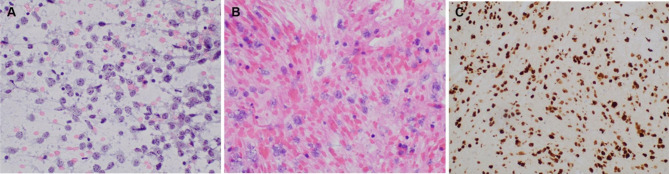
FNA of thyroid nodule (Case 3) showing abnormal population of large lymphocytes with prominent nucleoli and scant cytoplasm (A) (Pap stain, 400x magnification). Similar tumor cells were identified on the cell block (B) (H&E, 400 x magnification) with 90% positivity for Ki-67 (C) (200x magnification)

Ultrasound-guided FNA with ROSE of the left thyroid nodule was performed. The aspirate smears were abundantly cellular and contained predominantly large lymphocytes with irregular nuclear contours, prominent nucleoli, and scant cytoplasm (Fig. 8A) Air dried smears dedicated for FISH analysis were prepared and aspirated material was collected into separate RPMI media for both flow cytometry and cell block IHC. Flow cytometry studies demonstrated a CD10 positive and kappa light chain restricted population of B-cells. The cell block showed similar tumor cells to those seen on the aspirate smears (Fig. 8B). IHC performed on the cell block showed positive Ki67 (MIB-1) (K2, Leica) in 90% of cells (Fig. 8C) and negative staining for Myc (Y69, Abcam), BCL-2 Oncoprotein (124, Ventana), EBER ISH (ISH5687-A, Leica) and Cyclin D1 (SP4-R, Ventana).

FISH analysis showed no rearrangement of the *MYC* locus, although there was an extra copy of *MYC* and *IGH.* The diagnosis of DLBCL, germinal center type, was made with FNA alone.

## Discussion

Classically, the diagnosis of lymphoma has been based on histologic findings from surgically excised specimens, however, in many institutions FNA and CNB have become the primary diagnostic procedures. Controversy exists to whether a patient’s diagnosis of NHL can solely rely on results from FNA. A systematic review of 42 studies of FNA and CNB for the diagnosis of lymphoma performed by Frederiksen et al. in 2010 concluded that 25–35% of FNA and/or CNB of nodes required follow up with excisional biopsy to fully classify the lymphoma. In this review, diagnostic accuracy of NHL with FNA was highly dependent on the expertise of the practitioner and the resources available at the medical center [11].

The difficulty in diagnosis with FNA compared to tissue biopsy is often due to the loss of histologic architecture. Specific examples where this may be a challenge include composite lymphoma, DLBCL mimicking follicular lymphoma, grading errors when both high- and low-grade areas are present, when high grade is present, and the need to always exclude Burkitt lymphoma. Hehn et al. in 2010 revealed that FNA with the use of ancillary techniques including IHC or flow cytometry had a significantly greater correlation with excisional biopsy compared to FNA alone; however, these techniques were used by less than half of the pathology departments evaluated [12]. Therefore, at the time of this prior publication, the routine use of FNA for the diagnosis of lymphoma seemed infeasible at many institutions.

In the last decade, progress has been made to address this challenge. In 2014, Zhang et al. performed a study assessing the use of cell blocks as an adjunct to FNA for the diagnosis of lymphoproliferative disorders. This technique provided additional sample for IHC as well as improved assessment of tissue architecture. The addition of cell blocks improved diagnosis with a sensitivity of 99.0% and specificity of 95.9% for the discrimination of primary lymphoma, recurrent lymphoma, and benign reactive hyperplasia. In this study, the diagnostic accuracy of FNA with cell block was 87.5% (77/88) for NHL. As for B-cell NHL, this study revealed a high accuracy for subclassification of DLBCL, however, the diagnosis of mucosa-associated lymphoid tissue lymphoma with FNA remained challenging [9]. Similarly, in Patient 3 of our series, the diagnosis of DLBCL was made with ancillary testing performed on FNA material only, including cell block preparation for IHC, flow cytometry, and air-dried smears for FISH analysis.

A common presentation of thyroid lymphoma as a rapidly growing mass can render significant complications to tissue biopsy. The use of FNA material with cell block IHC provides a reliable alternative to CNB and incisional biopsy [13, 14]. FNA material alone has the potential to fully classify large B-cell lymphomas, the most common subtype of thyroid lymphomas, providing material for the necessary ancillary techniques including IHC, flow cytometry and FISH. The described diagnoses of lymphoma with CNB and incisional biopsy in our case series could have been made with FNA alone, as illustrated in Patient 3, based on the resources available at our institution. The proposed benefit of this technique is highlighted in Patient 1 where intubation for incisional biopsy proved to be difficult. Endotracheal intubation was successful, however, had this been unsuccessful, establishing a surgical airway would have been challenging given the presence of the thyroid mass. Although FNA may not be a widely accepted or available method for the diagnosis of lymphoma, if an institution has the resources and expertise available, this method should be considered as an initial diagnostic tool, particularly in this setting because it is safer and more cost-effective compared to core needle and open biopsy. The development of FNA procurement protocols for lymphoma can be helpful to ensure obtainment of adequate material needed for ancillary testing.

## Data Availability

Data was collected from patient charts during clinical encounters with authors listed and made unidentifiable for review and conception of manuscript.
